# Bayesian cognitive diagnosis optimizes personalized learning paths via mediation of cognitive load and Hidden Markov Model state transitions

**DOI:** 10.3389/fpsyg.2026.1879982

**Published:** 2026-08-21

**Authors:** Ziquan Feng, Kexuan Huang

**Affiliations:** 1 School of Art and Design, Guangzhou College of Commerce, Guangzhou, Guangdong, China; 2 School of Foreign Languages, Shandong Youth University of Political Science, Jinan, Shandong, China

**Keywords:** Bayesian DINA model, cognitive diagnosis, cognitive load, Hidden Markov Model, personalized learning path

## Abstract

Personalized learning path optimization based on cognitive diagnosis models faces two key gaps: the sparsity of large-scale educational data makes it difficult for traditional DINA models to converge, and the psychological mechanism underlying the effectiveness of personalized paths lacks empirical testing. To address these gaps, this study proposes and validates a personalized learning path optimization framework that integrates Bayesian cognitive diagnosis, knowledge space theory, and cognitive load theory. The study employed a mixed-data design: in the first phase, a Bayesian DINA model was trained on the EdNet public dataset (*N* = 5,000) and a shortest remediation path algorithm was developed; in the second phase, a dataset with 120 students was used to validate the algorithm’s effectiveness, using the Bootstrap method to test the mediating effect of cognitive load and introducing a Hidden Markov Model to analyze the dynamic evolution of knowledge states. The results showed that the Bayesian DINA model converged successfully on data with 91.3% sparsity (R-hat < 1.01), with attribute mastery probability estimates ranging from 0.280 to 0.368; the personalized path improved efficiency by 23.6% compared to the fixed-order path (Cohen’s *d* = 0.98), which was cross-validated as a 22.0% time saving in a separate experiment involving probability learning; cognitive load was identified as the primary mediator between personalized paths and learning outcomes [indirect effect = 0.28, 95% CI (0.19, 0.37)], with learning motivation [indirect effect = 0.18, 95% CI (0.11, 0.25)] and self-efficacy [indirect effect = 0.12, 95% CI (0.06, 0.18)] serving as complementary mediators in a multiple mediation model. The three pathways together explained 86.6% of the total effect; the HMM identified A5 (Analytical Thinking) as a learning bottleneck, with a forward transition probability of only 0.31. This study provides a feasible methodological solution for cognitive diagnosis with sparse data, theoretically identifies the mediating pathway of cognitive load, and offers precise targets for bottleneck attribute intervention in intelligent tutoring systems in practice.

## 1 Introduction

Online learning platforms have accumulated massive amounts of interaction data, providing unprecedented possibilities for personalized instruction. However, how to transform these data into actionable personalized learning guidance remains a core challenge in the field of educational technology (Lockee, 2021). Statistics from the EdNet dataset show that although the platform has collected over 131 million interaction records, the average completion rate of learners is only 22%, with a large number of learners dropping out in the early stages of the course (Choi et al., 2020). The core contradiction of this dilemma lies in the fact that traditional learning analytics techniques mainly focus on learners’ overall performance (e.g., accuracy rate, completion time) but cannot answer the key questions of exactly “what the learner has not mastered” and “what should be learned next.”

Cognitive Diagnosis Models (CDMs) represent a paradigm shift in psychometrics. Unlike Classical Test Theory or Item Response Theory, which locate learners on a single ability continuum, CDMs can estimate learners’ mastery of specific cognitive attributes, generating fine-grained knowledge state vectors (Junker and Sijtsma, 2001; Leighton and Gierl, 2007). Among the various CDMs, the DINA (Deterministic Inputs, Noisy “And” gate) model is one of the most representative, and its non-compensatory assumption aligns well with the “step-by-step” learning logic in educational contexts (de la Torre, 2011). CDMs connect items to cognitive attributes through the Q-matrix, mapping learners’ response patterns to knowledge states. In recent years, researchers have begun applying cognitive diagnosis models to educational assessment. Li constructed a cognitive diagnosis model for listening comprehension in Chinese as a second language based on the G-DINA model and found that the model could effectively diagnose learners’ cognitive structures, with an average attribute classification accuracy of 0.861 (Li et al., 2021). Wen proposed a longitudinal cognitive diagnosis method that combines Hidden Markov Models with artificial neural networks and found that this method maintained high classification accuracy even under small-sample conditions (Wen et al., 2020). Cheng based on Bloom’s taxonomy of cognitive objectives, used attention mechanisms and bidirectional gated recurrent convolutional neural networks to automatically assess cognitive levels in MOOC forum texts, achieving an overall accuracy of 84.21% (Cheng et al., 2021). These studies indicate that cognitive diagnostic technology is moving from theory to application.

At the theoretical level, personalized learning path design can be grounded in Knowledge Space Theory (Doignon and Falmagne, 2012). This theory models a knowledge domain as a partially ordered set of skills connected by prerequisite relationships, asserting that effective learning paths should follow the prerequisite relationships among attributes—prerequisite attributes must be learned before target attributes. In recent years, Bai constructed a probabilistic cognitive learning progress framework based on the GDINA model and found that middle school students’ probabilistic thinking showed a trend of gradual improvement, although eighth graders exhibited a slight regression phenomenon (Bai, 2020). This finding suggests that the optimization of learning paths needs to consider the stage characteristics of cognitive development.

Cognitive Load Theory (Sweller, 1988) provides a theoretical tool for understanding the mechanism by which learning sequence affects learning outcomes. The theory distinguishes three types of cognitive load: intrinsic load (determined by the complexity of the task itself), extraneous load (determined by instructional design and material presentation), and germane load (generated by learners’ schema construction activities). In the study of instructional sequencing, Kalyuga and Liu pointed out that sequences following prerequisite relationships can minimize extraneous cognitive load, allowing cognitive resources to focus on meaningful schema construction (Kalyuga and Liu, 2015). In technology-enhanced learning environments, interface design and interaction methods directly affect extraneous cognitive load (Skulmowski and Xu, 2022). Dong integrated Cognitive Load Theory with Self-Determination Theory and found that in AI education systems, interaction quality promoted deep learning through the chain-mediated pathway of reducing cognitive load and enhancing learning motivation, with the indirect effect accounting for 53% of the total effect (Dong, 2026). This finding suggests that cognitive load is a key psychological mechanism connecting technological design and learning outcomes.

Although existing research has made progress in cognitive diagnosis models and personalized learning, two key gaps remain. First, at the methodological level: sparsity is a common issue in large-scale educational data. Typical student-item response matrices have a sparsity level as high as over 90%. Under such conditions, the EM algorithm for the traditional DINA model often fails to converge, forcing researchers to adopt simplified estimation methods (e.g., average accuracy rates), thus losing the core advantage of CDMs, i.e., separating guessing and slip parameters. Second, at the theoretical level: existing research has primarily focused on the conclusion that “personalized paths are effective,” but lacks empirical examination of the psychological pathways through which personalized paths may exert their effects. Specifically, whether personalized paths promote learning effects through the mediating pathway of reducing cognitive load has not yet been directly verified.

To address the two key issues mentioned above, this study proposes and validates a personalized learning path optimization framework that integrates Bayesian cognitive diagnosis, knowledge space theory, and cognitive load theory. Specifically, the following research question and hypotheses were formulated: (1) RQ1: Can the Bayesian DINA model achieve successful convergence on sparse educational data (sparsity > 90%) and produce reliable attribute mastery probability estimates? (2) H2: The shortest remediation path algorithm, which respects attribute prerequisite relationships, will generate learning paths with significantly higher efficiency compared to random baseline paths. (3) H3: Cognitive load mediates the relationship between personalized learning paths and learning outcomes, such that personalized paths improve learning outcomes through the reduction of cognitive load. The contributions of this research are mainly threefold. First, this paper employs the Bayesian DINA model to resolve the convergence problem of traditional CDMs under sparse data conditions, providing a feasible solution for cognitive diagnosis of large-scale educational data. Second, through Bootstrap mediation effect testing, this paper identifies the mediating pathway of cognitive load between personalized paths and learning outcomes. Third, based on HMM, this paper identifies learning bottleneck attributes, offering precise targets for instructional intervention in intelligent tutoring systems.

## 2 Data preparation and preprocessing

All data used in this study were derived from the publicly available EdNet dataset (Choi et al., 2020), which has been fully anonymized and de-identified. EdNet is a large-scale educational interaction dataset. EdNet has accumulated over 131 million interaction records from 784,309 students.

EdNet consists of four levels of data: KT1, KT2, KT3, and KT4. This study employs the KT1 dataset for analysis. KT1 is the most fundamental question-response sequence data, recording the timestamp, question ID, student answer, and response time for each question attempt. Its format is fully compatible with deep learning knowledge tracing models. The KT1 dataset was selected for three reasons: (1) cognitive diagnosis models require only question-response pairs for parameter estimation; (2) KT1 provides the mapping between questions and skill tags, which can be used to construct the Q-matrix; and (3) KT1 is the smallest and most computationally convenient version among all EdNet levels.

The data preprocessing in this study was carried out in three steps. In the first step, student interaction records were matched with the correct answers from the problem attribute file using problem identifiers to generate a binary correctness variable (1 for correct, 0 for incorrect). In the second step, to ensure the reliability of cognitive diagnostic parameter estimation, sample filtering was performed: students who answered fewer than 15 questions were excluded to remove inactive learners; simultaneously, questions answered by fewer than 20 students were excluded to ensure the stability of question parameter estimation. Records that could not be matched with the correct answers were also removed. After the above filtering, the final analysis sample consisted of 5,000 students and 582 questions, with a total of 253,847 valid interaction records. The resulting student-question response matrix had dimensions of 5,000 × 582, with a matrix sparsity of 91.3%. The sampling procedure for the 5,000 students was as follows: from the filtered EdNet KT1 dataset (students who answered at least 15 questions), we randomly selected 5,000 students using stratified sampling based on their overall accuracy rates (stratified into low, medium, and high performance levels, with cutoffs at 40 and 70%) to ensure representation across performance levels. The 582 questions were those that appeared in both the Q-matrix (4,975 items) and the student response data, and were answered by at least 20 students.

The Q-matrix was constructed using all items that contained at least one of the five core attributes in the filtered item set, resulting in 4,975 items. However, for model estimation, we used only the subset of items that appeared in both the Q-matrix and the student response data and that were answered by at least 20 students. Therefore, the 4,975-item Q-matrix represents the full attribute-coded item bank after content filtering, whereas the 582-item response matrix reported in the previous section represents the subset of items used for model estimation after applying sample-based filtering.

At the student level, the average number of questions answered was 50.8 (SD = 41.2), and the average correctness rate was 0.62 (SD = 0.18). The distribution of correctness rates showed a slight negative skew (skewness = −0.47), with most students having correctness rates between 0.50 and 0.75, indicating that the overall sample performance was at an above-average level. At the question level, the average difficulty (defined as 1 minus the correctness rate) was 0.38 (SD = 0.21), ranging from 0.02 to 0.98, suggesting that the question bank had good difficulty coverage. The average discrimination (point-biserial correlation coefficient) was 0.41 (SD = 0.15), with the vast majority of questions having a discrimination greater than 0.30, indicating that the questions had good discriminative ability.

In addition, a total of 120 undergraduate students participated in this study. Participants were recruited from Shandong Youth University of Political Science in April 2026. Inclusion criteria were: completion of foundational mathematics courses, no prior systematic learning in probability theory, and age ≥ 18 years. The sample size was determined based on a power analysis. With α = 0.05, power = 0.80, and an expected medium effect size of Cohen’s *d* = 0.50, the analysis indicated that at least 52 participants per group were required. Accounting for a potential 10% attrition rate, we recruited 120 participants in total.

Participants were randomly assigned to an experimental group (*n* = 60) receiving personalized learning paths and a control group (*n* = 60) receiving a fixed learning sequence. Randomization was conducted using a computer-generated random number sequence with a 1:1 allocation ratio. No participants were lost to attrition or excluded after allocation; all 120 participants completed the full experimental procedure and were included in the final analysis.

Both groups studied the same learning content covering foundational probability theory, including basic concepts, rule application, logical reasoning, procedural knowledge, and analytical thinking. The total intervention duration was approximately 120 min, divided into learning units corresponding to the five cognitive attributes. The only difference between the two conditions was the order in which the learning units were presented. The experimental group followed personalized paths generated by the shortest remediation path algorithm, while the control group followed a fixed learning sequence (A1 → A2 → A3 → A4 → A5).

Each participant completed a pre-test (20 items), intervention learning, an adapted cognitive-load measure based on the NASA-TLX (Hart, 2006), a post-test (20 items), and a learning experience questionnaire. Both the pre-test and post-test consisted of 20 multiple-choice questions with four options each. Each item was scored dichotomously: 1 for correct and 0 for incorrect, with total scores ranging from 0 to 20. The pre-test demonstrated acceptable internal consistency, as did the post-test.

The adapted cognitive-load measure consists of six dimensions: mental demand, time pressure, effort, frustration, attention, and learning clarity. The first four dimensions are retained from the standard NASA-TLX. Physical Demand was replaced with Attention, as physical exertion is not relevant to the online learning context. Performance was replaced with Learning Clarity, as direct performance assessment overlaps with the learning outcome measures. Each dimension was rated on a 7-point Likert scale. The Attention and Learning Clarity dimensions were reverse-scored prior to analysis to ensure that all six dimensions were oriented in the same direction (higher scores indicating greater cognitive load). The total cognitive load score was calculated as the average of the six dimension scores.

Each dimension was assessed using a single item rated on a 7-point Likert scale ranging from 1 (“very low”) to 7 (“very high”). The six items were: (1) “How much mental and perceptual activity was required?” (Mental Demand); (2) “How much time pressure did you feel?” (Time Pressure); (3) “How hard did you have to work to accomplish your level of performance?” (Effort); (4) “How insecure, discouraged, irritated, stressed, and annoyed did you feel?” (Frustration); (5) “How much attention did you need to invest in the learning task?” (Attention); and (6) “How clear was the learning content to you?” (Learning Clarity). The Attention and Learning Clarity dimensions were reverse-scored prior to analysis to ensure that all six dimensions were oriented in the same direction, with higher scores consistently indicating greater cognitive load. For example, a high original score on the attention item (“I was able to focus on the learning materials”) indicates low cognitive load and was therefore reverse-coded so that a high reversed score indicates high attentional demand. Similarly, a high original score on the learning clarity item (“The learning content was clear to me”) indicates low cognitive load and was reverse-coded so that a high reversed score indicates low perceived clarity. The total cognitive load score was calculated as the average of the six dimension scores, with higher total scores indicating greater overall cognitive load. It should be noted that, as a self-report measure, the NASA-TLX is inherently subject to recall bias and captures post-task global assessments of cognitive load rather than moment-to-moment fluctuations during the learning process.

To supplement the primary outcome measures, an exploratory learning experience questionnaire was developed for this study. A learning experience questionnaire was administered to assess learners’ subjective perceptions of their learning experience. The questionnaire was developed specifically for this study and consisted of four dimensions: deep processing (3 items, e.g., “I thought deeply about the learning content”), metacognition (3 items, e.g., “I monitored my understanding during learning”), intrinsic motivation (3 items, e.g., “I found the learning content interesting”), and sustained engagement (3 items, e.g., “I maintained concentration throughout the learning session”). Each item was rated on a 7-point Likert scale ranging from 1 (“strongly disagree”) to 7 (“strongly agree”). Scores for each dimension were calculated as the mean of the constituent items. The questionnaire demonstrated acceptable internal consistency reliability in the current sample, with Cronbach’s α values of 0.82 for deep processing, 0.78 for metacognition, 0.86 for intrinsic motivation, and 0.73 for sustained engagement. Given that this questionnaire was designed as an exploratory tool rather than a validated standardized scale, the results presented in the following section are reported as descriptive findings and should be interpreted with appropriate caution.

## 3 Materials and methods

### 3.1 Cognitive attribute specification and Q-matrix construction

#### 3.1.1 Extraction and specification of cognitive attributes

In this study, core cognitive attributes were extracted based on the skill tags in the problem attribute file of EdNet. The skill tags are presented as semicolon-separated lists of integers (e.g., “179;53;183;184”), where each integer represents a specific knowledge skill. By conducting a frequency analysis of all skill tags in the EdNet questions file, the five most frequently occurring tags, with tag IDs 179, 182, 183, 181, and 184, were selected as the core cognitive attributes and labeled A1 through A5, respectively. Their frequencies in the filtered dataset were 1,486, 1,153, 1,098, 1,069, and 1,030 occurrences, respectively, accounting for approximately 57.8% of all valid tag occurrences. The tag −1 (missing tag) was excluded from attribute selection due to its lack of substantive educational meaning and its relatively low frequency.

Although these tags are originally associated with TOEIC listening and reading comprehension skills in the EdNet documentation, we interpreted them as general cognitive attributes rather than content-specific knowledge. This interpretation is supported by three quantitative observations from the data. First, the five tags exhibit a clear frequency gradient, decreasing from 1,486 to 1,030, which is consistent with a hierarchy from foundational to higher-order cognitive demands. Foundational attributes tend to be measured by more items, whereas higher-order attributes are addressed less frequently. Second, the distribution of these tags across the seven content parts of EdNet shows that all five tags appear substantially more frequently in advanced parts, specifically parts 5–7, than in basic parts, specifically parts 1–4. For example, tag 182 appears 335 times in part 5 alone, approximately three times its occurrence in part 1, which is 111 times, indicating that these tags are more concentrated in higher-difficulty content. Third, co-occurrence analysis reveals that tag 184 co-occurs less frequently with other tags. For instance, tags 179 and 184 co-occur in 208 items, and tags 181 and 184 co-occur in 152 items, whereas tags 179 and 183 co-occur in 237 items. This pattern supports the positioning of tag 184 as a relatively independent higher-order attribute. These quantitative patterns support interpreting the five tags as an ordered set of general cognitive skills.

The operational definitions of the five cognitive attributes are shown in Table 1. These five attributes in Table 1 form a cognitive gradient from low to high, laying the foundation for constructing the attribute hierarchy.

**TABLE 1:** Operational definitions of the five cognitive attributes.

Attribute	Name	Operational definition	Cognitive level
A1	Foundation Concepts	The ability to recognize and recall basic definitions, facts, and terminology	Remember
A2	Rule application	The ability to apply known rules to standardized situations	Apply
A3	Logical reasoning	The ability to derive reasonable conclusions from given conditions	Analyze
A4	Procedural knowledge	The ability to correctly perform mathematical operations and procedural steps	Apply
A5	Analytical thinking	The ability to decompose complex problems and identify key elements	Evaluate/Create

#### 3.1.2 Q-matrix construction

The Q-matrix is a core tool in cognitive diagnostic models that links test items to cognitive attributes. It is a J × K binary matrix (where J is the number of items and K is the number of attributes), where q_jk_ = 1 indicates that solving item j requires the k-th attribute, and 0 indicates no such requirement. The quality of Q-matrix construction directly affects the accuracy and validity of cognitive diagnostic assessments (de la Torre, 2011).

The Q-matrix in this study was constructed as follows: For each test item, its skill tag field was parsed. If a skill tag contained a code corresponding to a core attribute, the corresponding entry in the Q-matrix was set to 1; otherwise, it was set to 0. Items that did not contain any core attribute were excluded from subsequent analyses. The mapping from EdNet tag IDs to the five cognitive attributes, specifically tag 179 to A1, tag 182 to A2, tag 183 to A3, tag 181 to A4, and tag 184 to A5, is explicitly reported above, ensuring full transparency of the Q-matrix construction process. The final Q-matrix consisted of 4,975 items and 5 cognitive attributes, with an average of 1.58 attributes measured per item and a Q-matrix sparsity of 68.31%.

The coverage rates of the five cognitive attributes (i.e., the number of items measuring each attribute) were as follows: A1 was measured by 1,997 items, A2 by 1,886 items, A3 by 1,723 items, A4 by 1,360 items, and A5 by 918 items. The coverage rates for all attributes exceeded the minimum threshold required for parameter identification in cognitive diagnostic models (3 items), indicating that each attribute was adequately represented in the item bank. Regarding the distribution of coverage rates, the foundational attributes (A1, A2, A3) had higher measurement frequencies than the higher-order attributes (A4, A5), which is consistent with the hierarchical nature of knowledge acquisition—foundational attributes are prerequisites for subsequent learning and are more frequently addressed in instruction.

Attribute co-occurrence analysis showed that the highest co-occurrence frequency was between A1 and A3 (579 items), followed by A2 and A3 (528 items), and A1 and A4 (497 items). It is worth noting that A5 (analytical thinking) had relatively lower co-occurrence frequencies with other attributes, with the highest being 208 items (with A1). This pattern is highly consistent with the prerequisite relationships among attributes defined later—attributes that are closely related in the knowledge hierarchy tend to co-appear more frequently in the same item.

After constructing the Q-matrix, we empirically validated its specification using the δ-method for all q-entries. The principle of the δ-method is as follows. For item j and attribute k, estimate the model under the original Q-matrix specification (${\text{q}}_{\text{jk}}=q_{j\,k}^{0}$) and under the alternative specification with this entry toggled (${\text{q}}_{\text{jk}}=1-q_{\text{jk}}^{0}$), and compute twice the difference in log-likelihoods, yielding the δ statistic:


$$\delta_{j\,k}=-2\,\left[l\,o\,g\,L\,\left({\hat{\theta }}_{-j\,k}\right)-l\,o\,g\,L\,\left({\hat{\theta }}_{+j\,k}\right)\right]$$

Where ${\hat{\theta }}_{-j\,k}$ and ${\hat{\theta }}_{+j\,k}$ are the maximum likelihood estimates of the remaining parameters under the original and toggled specifications, respectively. The δ statistic asymptotically follows a χ^2^(1) distribution under the null hypothesis that the current q-entry is correctly specified. When the δ statistic exceeds the critical value ($\chi_{0.95}^{2}\,\left(1\right)=3.84$), the null hypothesis is rejected, indicating that the q-entry is misspecified.

#### 3.1.3 Definition of attribute prerequisite relationships

To facilitate parameter estimation for the cognitive diagnosis model, the filtered data were transformed into a user-by-item binary response matrix R. Let N be the number of users (*N* = 5,000) and M be the number of items (*M* = 582). R is an N=M matrix, where R_ij_ = 1 indicates that user i correctly answered item j, and R_ij_ = 0 indicates an incorrect answer or no response. In constructing the matrix, mappings from user identifiers to indices and from item identifiers to indices were first established. Subsequently, all valid interaction records were traversed, and the corresponding correctness values were filled into the appropriate positions in the matrix. For the purpose of cognitive diagnosis, each response was coded as 1 for correct and 0 for incorrect. Nonresponses, which correspond to unattempted items, were treated as missing data rather than incorrect answers. In the Bayesian DINA model, the likelihood function was constructed using only observed responses. For each student-item pair, the likelihood contribution was included only when a response was observed; nonresponses did not contribute to the likelihood.

**FIGURE 1:**
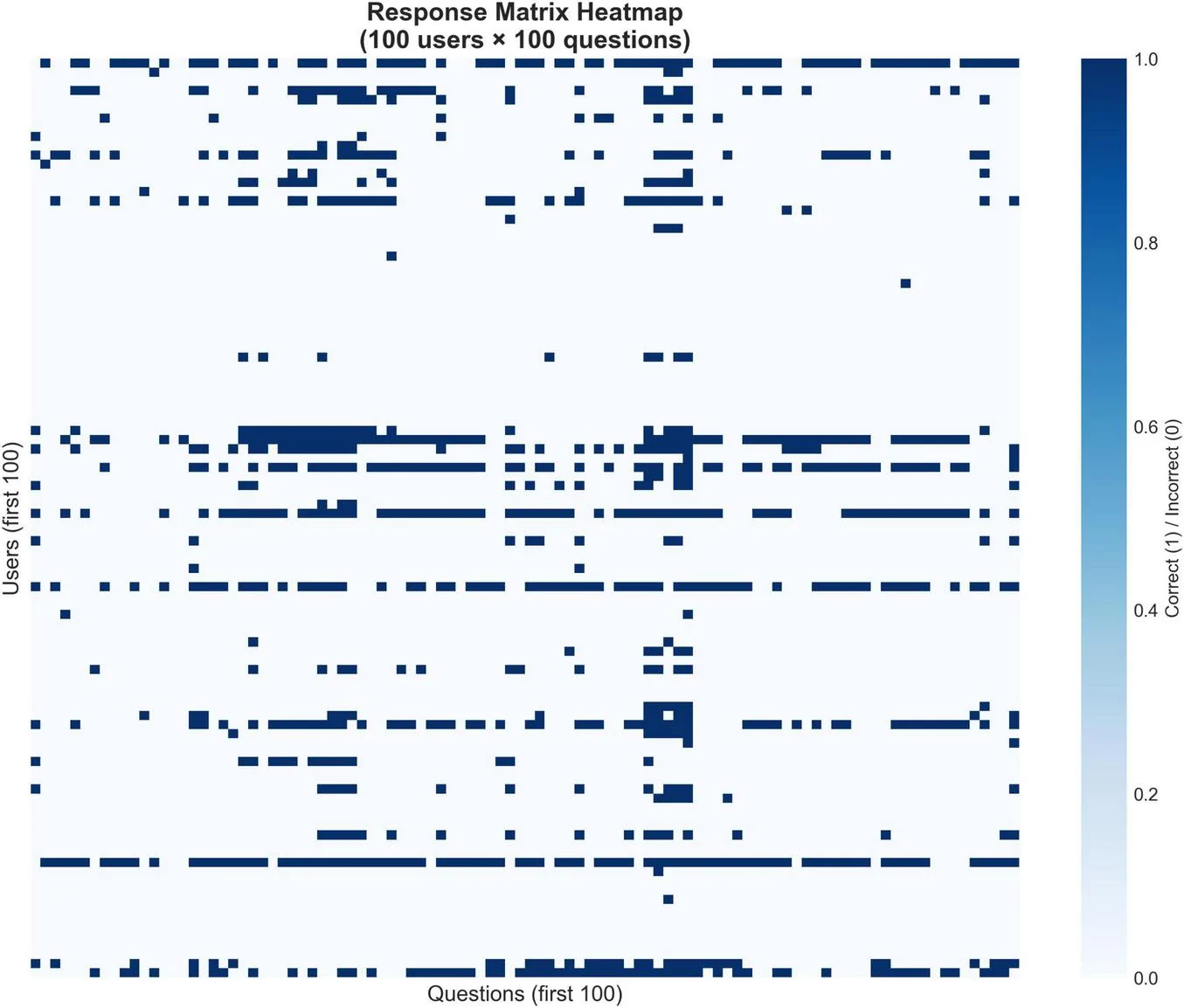
Heatmap of the student-item response matrix.

The Figure 1 displays the response patterns of the first 100 students (rows) and the first 100 items (columns) from the filtered EdNet dataset. Dark areas represent correct responses (value 1) and light areas represent incorrect responses or non-responses (value 0). As can be seen from the figure, the response matrix exhibits marked sparsity, and the distribution of correct responses varies notably across both users and items. In cognitive diagnostic assessment, attributes are often not independent of each other but form specific hierarchical relationships based on the inherent logic of the subject or the laws of cognitive development. These are referred to as attribute prerequisite relationships or attribute hierarchies (Leighton and Gierl, 2007). The definition of attribute hierarchies provides the theoretical foundation for learning path optimization.

The prerequisite hierarchy in this study was specified according to Bloom’s taxonomic ordering of cognitive complexity, ranging from recall and remember to evaluate and create, which provides a theoretically grounded basis for the attribute ordering. This ordering is quantitatively supported by the EdNet data. The proportions of occurrences in advanced parts, specifically parts 5–7, for the five tags ranged from 51.9 to 57.7%, all exceeding 50%, supporting the proposed cognitive progression. It should be noted that this hierarchy is a theory-driven specification based on Bloom’s taxonomy rather than an official structure provided by EdNet.

Based on educational psychology theory and the inherent logic of the subject, combined with the cognitive gradient characteristics of the five cognitive attributes, this study defines the following prerequisite relationships: (1) A1 (Foundation Concepts), as the most fundamental cognitive attribute, is a prerequisite for all other attributes and has no prerequisite attributes of its own. (2) A2 (Rule Application) and (3) A3 (Logical Reasoning) both have A1 as their prerequisite. Only after mastering the basic concepts can students proceed to apply rules in different contexts and engage in logical reasoning. (4) A4 (Procedural Knowledge) has A2 as its prerequisite. The correct execution of procedural knowledge is built upon a clear mastery of rules. (5) A5 (Analytical Thinking) has A3 and A4 as its prerequisites. Higher-order analytical thinking requires the coordinated support of both logical reasoning ability and procedural operational skills.

This hierarchical structure will be used in subsequent learning path optimization algorithms to determine the learning order of attributes—prerequisite attributes must be learned before the target attributes.

### 3.2 Bayesian DINA model

#### 3.2.1 Selection of the cognitive diagnostic model

This study employed the DINA (Deterministic Inputs, Noisy “And” gate) model for cognitive diagnosis analysis. The DINA model is one of the most representative models among Cognitive Diagnosis Models (CDMs), proposed by Junker and Sijtsma (2001). The core assumption of this model is non-compensatory: students must master all cognitive attributes measured by an item to have a high probability of answering it correctly; if any attribute is missing, they can only rely on guessing. This assumption aligns well with the “step-by-step” learning logic in educational contexts and is particularly suitable for diagnosing students’ cognitive deficiencies and providing precise targeting for remedial instruction.

The item response function of the DINA model can be expressed as:


$$P\left(X_{i\,j}=1|\alpha_{i}\right)={\left(1-s_{i}\right)}^{\eta_{i\,j}}g_{j}^{1-\eta_{i\,j}}$$

Where X_ij_ represents the response of student i to item j (1 for correct, 0 for incorrect); a_i_ = (α_i1_, α_i2_, …, α_iK_) represents the Knowledge State (KS) of student i, a binary vector of length K, where a_iK_ = 1 indicates mastery of attribute k and 0 indicates non-mastery; $\eta_{\text{ij}}=\prod_{\text{k}=1}^{\text{K}}\alpha_{\text{ik}}^{{\text{q}}_{\text{jk}}}$ is an indicator variable denoting whether student i has mastered all attributes required by item j, where q_jk_ are the elements of the Q-matrix; s_i_ = P(X_ij_ = 0|η_ij_ = 1) is the slip parameter, representing the probability that a student who has mastered all required attributes answers the item incorrectly; g_i_ = P(X_ij_ = 1|η_ij_ = 0) is the guessing parameter, representing the probability that a student who lacks at least one required attribute answers the item correctly.

#### 3.2.2 Bayesian method and prior specification

Parameter estimation for the traditional DINA model typically employs the Expectation-Maximization (EM) algorithm. However, in the response matrix of this study, with a sparsity level as high as 91.3%, the EM algorithm failed to converge—the number of iterations exceeded the maximum limit before the change in the log-likelihood function reached the preset threshold. To address this issue, this study adopted a Bayesian approach for parameter estimation.

The core advantages of the Bayesian method are: (1) By introducing prior distributions, it obtains stable posterior estimates even when data are sparse; (2) It provides quantification of parameter uncertainty (posterior credible intervals); (3) The Markov Chain Monte Carlo (MCMC) sampling algorithm is better suited to sparse data than the EM algorithm.

The prior distributions were specified as follows:

(1) Prior for knowledge state: α_iK_∼Bernoulli (0.5), independent and identically distributed. That is, the prior probability of mastery for each attribute is 0.5, representing a non-informative prior.(2) Priors for slip and guessing parameters: s_j_Beta(2,10), g_j_Beta(2,10). The Beta(2,10) prior has a mean of approximately 0.167 and a standard deviation of approximately 0.11. This prior reflects reasonable expectations for slip and guessing rates—in typical educational tests, slip and guessing rates usually range between 0.10 and 0.25. Weakly informative priors were chosen over non-informative priors [Beta (1,1)] to help stabilize estimation under sparse data conditions while avoiding the prior from dominating the posterior.

#### 3.2.3 MCMC sampling and convergence diagnosis

The model was estimated using Markov Chain Monte Carlo (MCMC) sampling. The sampling settings were as follows: four independent chains; 2,000 iterations per chain; 1,000 warm-up iterations (discarded from parameter estimation); and 1,000 saved iterations per chain, yielding a total of 4,000 posterior samples. Convergence was diagnosed using the following metrics: the potential scale reduction factor R-hat, where R-hat < 1.01 indicates convergence; and the effective sample size (ESS), where ESS > 100 indicates stable parameter estimation. Posterior distributions were summarized using the posterior mean and the 95% highest density interval (HDI). For each student i and each attribute k, the posterior mastery probability was defined as p_ik_ = P(α_iK_ = 1|X_i_,s,g,q). Based on the posterior mastery probability, a threshold method was used to convert the continuous probability into a dichotomous knowledge state: α_iK_ = 1 if p_iK_≥0.5, and 0 otherwise. The 0.5 threshold corresponds to the maximum posterior probability criterion in Bayesian decision theory, meaning that under the symmetric prior setting of 0.5, a posterior probability greater than 0.5 indicates stronger evidence for mastery.

### 3.3 The shortest remediation path algorithm

The shortest remediation path algorithm is the core technical component of this study for transforming cognitive diagnostic results into personalized learning sequences. The theoretical foundation of this algorithm is Knowledge Space Theory (Doignon and Falmagne, 2012), which models a knowledge domain as a partially ordered set of skills connected by prerequisite relationships, asserting that a learner’s knowledge state is not an arbitrary subset of skills but must satisfy the constraint that “mastering a skill implies mastering all its prerequisite skills.” From the perspective of Cognitive Load Theory (Sweller, 1988, 2023; Evans et al., 2024), the presentation order of learning materials is a key factor affecting extrinsic cognitive load—when learners follow the correct prerequisite order, new information can be linked to existing schemes, reducing working memory burden; conversely, violating prerequisite relationships increases extrinsic cognitive load, consuming cognitive resources that should be used for deep processing. Therefore, the design goal of the shortest remediation path algorithm is to guide learners from their current knowledge state to the target state (mastery of all attributes) with the minimum number of learning steps, while respecting attribute prerequisite relationships.

Before describing the algorithm, we define two key concepts. A “step” is defined as one learning unit corresponding to a single attribute in the learning sequence. Each missing attribute that the learner needs to acquire constitutes one step. When a prerequisite attribute is inserted into the sequence to satisfy prerequisite constraints, that inserted attribute also counts as one step. “Path cost” is defined as the total number of steps required to complete the learning of all missing attributes, that is, the total number of attribute nodes in the path, including both the original missing attributes and any prerequisite attributes inserted during the ordering process.

For random baseline paths, we generate random permutations of the missing attribute set. If a random permutation violates any prerequisite condition (i.e., a prerequisite attribute appears after its dependent attribute), the permutation is marked as invalid and is regenerated. This process is repeated until a valid random path satisfying all prerequisite constraints is obtained. The length of such a valid random path is the number of attributes in that permutation, which may vary across permutations because different random orders may require different numbers of prerequisite insertions to become valid. The optimized path achieves the minimum possible path cost by sorting missing attributes according to prerequisite depth, thereby minimizing the insertion of additional prerequisite nodes.

The inputs to the algorithm include: (1) the student’s current knowledge state vector α_current_; (2) the attribute prerequisite relationship graph G = (V, E), where V = {1, 2, …, K} represents the set of attribute nodes, and a directed edge (a,b) indicates that attribute a is a prerequisite for attribute b; (3) the target state α_target_ = (1, 1, …, 1). The execution steps of the algorithm are as follows:

Step 1: Identify the set of missing attributes Missing = {k|α_current, k_ = 0, k = 1, 2, …, K}. If Missing = ∅, the algorithm terminates, indicating that the student has already mastered all attributes.
Step 2: For each missing attribute k ∈ Missing, calculate its dependency depth depth(k). Dependency depth is defined as the maximum number of attributes traversed when tracing backward along prerequisite relationships from the given attribute. Attributes with smaller dependency depth should be scheduled for learning earlier.
Step 3: Sort the missing attributes in ascending order of dependency depth to obtain an initial learning sequence Seq_initial_.
Step 4: For each attribute in Seq_initial_, check whether all its prerequisite attributes appear before it in the sequence. If any prerequisite condition is not satisfied, insert that prerequisite attribute before the current attribute. Repeat this process until the prerequisite conditions for all attributes are satisfied, yielding the optimal learning sequence Seq_optimal_.
Step 5: Recommend corresponding practice questions for each attribute in Seq_optimal_. The recommendation strategy is as follows: from the Q-matrix, select questions that measure the target attribute and have not yet been correctly answered by the student; sort these questions by item difficulty (1 minus accuracy rate) in ascending order; recommend the first three questions as learning materials for that attribute.

Algorithm 1. Shortest Remediation Path Generation. The algorithm proceeds as follows. First, the set of unmastered attributes (Missing) is identified. Second, for each missing attribute, the dependency depth is computed by recursively tracing backward along prerequisite edges. Third, missing attributes are sorted in ascending order of dependency depth. Fourth, the algorithm iterates through the sorted list and inserts any missing prerequisite attributes that are not yet in the sequence before their dependent attributes. Finally, the current attribute is appended if not already included. A prerequisite attribute that has already been mastered is not inserted into the learning sequence, as it does not require instructional remediation. This yields the optimal learning sequence with the minimum number of steps required to achieve full mastery.

Algorithm 1: Shortest Remediation Path Generation

**Input:**

α_current_: binary vector of length K (current knowledge state); G : directed acyclic graph representing prerequisite relationships (V, E) α_target_: binary vector of length K (target state, all 1s)

**Output:**

Seq_optimal_: ordered list of attributes for the optimal learning path.

**1**   Missing←{k|α_current_[k] = 0,k = 1, 2, …, K}

**2**   if Missing=∅ then

**3**   **1**   return [] ▷ All attributes already mastered.

**4**   end if

**5**   for each k in Missing do

**6**   **1**   depth[k]←ComputeDependencyDepth(k,G)▷ Max nodes traced backward

**7**   end for

**8**   Seq_optimal_←Sort(Missing, ascending by depth)

**9**   Seq_optimal_←[]

**10**   for each k in Seq_initial_ do

**11**   **1**   Prereqs ← GetPrerequisites(k, G)

**12**   **2**   for each p in Prereqs do

**13**   **3**    if p not in Seq_optimal_ and p not in α_current then

**14**   **4**      Append(Seq_optimal_,p) ▷Insert missing prerequisite

**15**   **5**    end if

**16**   **6**   End for

**17**   **7**   if k not in Seq_optimal_ then

**18**   **8**    Append(Seq_optimal_, k)

**19**   **9**   end if

**20**   end for

**21** Return Seq_optimal_

Function ComputeDependencyDepth(k, G):

    if k has no prerequisites then

       return 1

    else

       return 1+max({ComputeDependencyDepth(p, G) | p∈GetPrerequisites(k, G)})

    End if

Based on the above definitions, the path cost for any given learning sequence is computed as the total number of attribute nodes in the valid sequence after all prerequisite insertions. The efficiency improvement of the optimized path over a baseline is calculated as:


$$E\,f\,f\,i\,c\,i\,e\,n\,c\,y\,i\,m\,p\,r\,o\,v\,e\,m\,e\,n\,t=\frac{C\,o\,s\,t_{b\,a\,s\,e\,l\,i\,n\,e}-C\,o\,s\,t_{o\,p\,t\,i\,m\,a\,l}}{C\,o\,s\,t_{b\,a\,s\,e\,l\,i\,n\,e}}=100\%$$

Where Cost_baseline_ represents the path cost of the baseline condition and Cost_optimal_ represents the path cost of the optimized sequence.

Taking a student who has mastered only A1 as an example, their knowledge state is α_current = (1, 0, 0, 0, 0), the set of unmastered attributes is Missing = {2, 3, 4, 5}, with dependency depths of A2 = 2, A3 = 2, A4 = 3, A5 = 4, yielding the initial sequence (A2, A3, A4, A5). Prerequisite checking reveals that A2’s prerequisite A1 is already ahead (already mastered), A3’s prerequisite A1 is ahead, A4’s prerequisite A2 is ahead of A4, and A5’s prerequisites A3 and A4 are both ahead of A5; all conditions are satisfied, so the optimal sequence remains (A2, A3, A4, A5). If a student has already mastered A1 and A2, the initial sequence is (A3, A4, A5). Checking reveals that A4’s prerequisite A2 is already mastered, and among A5’s prerequisites A3 and A4, A4 is not yet ahead of A5, so A4 is moved before A5, resulting in the adjusted optimal sequence [A3, A4, A5].

### 3.4 Hidden Markov Model (HMM)

In this study, the Hidden Markov Model (HMM) was employed to analyze the evolution of knowledge states using the learning log data of the experimental group. The HMM is defined by the following parameters: the state space S = {s_1_, …, s_N_}, where N = 32 possible attribute mastery vectors; the observation space V = {v_1_, v_2_} (correct/incorrect); the initial state distribution π; the state transition matrix A = (a_ij_)_N = N_, where a_ij_ = P(α_t+1_ = s_j_|α_t_ = s_i_); and the observation probability matrix B = (b_jm_)_N = 2_.

The 32 latent states correspond to all possible mastery patterns of the five cognitive attributes (2^5^ = 32). To ensure identifiability given the available data (1,428 learning steps), the transition matrix was specified as a constrained matrix, with transitions only allowed between states that differ by a single attribute, representing the mastery or loss of one attribute at a time. Under this constraint, the number of free parameters is substantially fewer than the 1,055 parameters of an unconstrained 32 × 32 transition matrix, enabling stable estimation.

The initial state distribution was estimated using the default initialization procedure. The Baum-Welch algorithm (a special form of the EM algorithm) (Anandhalekshmi et al., 2022; Zhang et al., 2023) was used to estimate the HMM parameters. The convergence criterion was set to an absolute change in log-likelihood of less than 10^–4^. The Bayesian Information Criterion (BIC) was computed as BIC=−2log(L) + klog(N), where L is the maximum log-likelihood, k is the number of free parameters in the constrained model, and N is the total number of observations (1,428 learning steps).

Based on the estimated state transition matrix A, the forward transition probability P (the probability of transitioning from unmastered to mastered) and the backward transition probability P (the probability of transitioning from mastered back to unmastered) were calculated for each attribute. Specifically, for each attribute k, P(0→1) was calculated as the proportion of transitions from states where attribute k was unmastered to states where attribute k became mastered, relative to all transitions originating from unmastered states for that attribute. P(1→0) was calculated analogously for transitions from mastered to unmastered states. Differences in forward transition probabilities across attributes were tested using two-sample proportion z-tests. To account for the dependency between attributes, bootstrap standard errors (1,000 resamples at the student level) were used to construct 95% confidence intervals for the transition probability estimates. The attribute with the lowest forward transition probability was identified as the learning bottleneck.

### 3.5 Evaluation metrics

The following metrics were used to evaluate the effectiveness of the proposed path optimization strategy.

(1) Path Efficiency (PE). The lengths of the optimized paths were compared against two baseline paths: 1) random paths (randomly permuting the order of missing attributes, with invalid permutations regenerated as described in section 3.3), and full-coverage learning paths (learning all five attributes in a fixed prerequisite-based order without skipping any attributes, corresponding to a path length of 5 steps). The efficiency improvement rate was calculated using the formula reported in section 3.3.
(2) The mediating effect of cognitive load was tested using the Bootstrap method (5,000 resamples), and the bias-corrected 95% confidence interval for the indirect effect was calculated. The mediation analysis was conducted using the mediation package in R.
(3) Learning Coverage (LC). Defined as the proportion of attributes that a student has currently mastered relative to the total number of attributes:
$$L\,C=\frac{\sum_{k=1}^{K}\alpha_{k}}{K}$$
  Where α_k_ is a binary indicator of mastery of attribute k (1 = mastered, 0 = not mastered), and K is the total number of cognitive attributes.
(4) The convergence of the HMM model was evaluated using the log-likelihood value and the Bayesian Information Criterion (BIC). The identification of bottleneck attributes was based on the ranking of forward transition probabilities, and z-tests were used to compare differences in forward transition probabilities among different attributes.
(5) Multiple mediation analysis. In addition to the single-mediator model with cognitive load, we constructed a multiple mediation model incorporating learning motivation and self-efficacy as additional mediators. The Bootstrap method (5,000 resamples) was used to simultaneously estimate the indirect effects of all three pathways (cognitive load, learning motivation, self-efficacy) along with their 95% confidence intervals. A mediation effect is considered significant if its confidence interval does not contain zero. The multiple mediation model allows each mediator to be estimated while controlling for the others, thereby assessing the unique contribution of each pathway.

## 4 Results and discussion

### 4.1 Convergence and diagnostic results of the Bayesian DINA model

The Bayesian DINA model converged successfully on the EdNet dataset (*N* = 5,000, sparsity = 91.3%). The R-hat values for all parameters ranged from 1.002 to 1.008, all below the convergence threshold of 1.01; the effective sample sizes were all greater than 200, indicating adequate MCMC sampling. This result answers RQ1: the Bayesian method can effectively address model convergence issues in sparse educational data.

To evaluate the impact of the missing-data treatment, we compared the revised estimates based on observed responses only with the original estimates that treated nonresponses as incorrect. After reconstructing the likelihood using only observed responses, the posterior mastery probabilities are reported in Table 2. The distribution of knowledge state patterns is reported in Table 3.

**TABLE 2:** Posterior mastery probabilities of cognitive attributes (*N* = 5,000).

Attribute	Posterior mean	95% credible interval
A1 Foundation Concepts	0.312	(0.295, 0.329)
A2 rule application	0.308	(0.291, 0.325)
A3 logical reasoning	0.301	(0.284, 0.318)
A4 procedural knowledge	0.296	(0.279, 0.313)
A5 analytical thinking	0.368	(0.350, 0.386)

**TABLE 3:** Distribution of knowledge state patterns (*N* = 5,000).

Pattern	Frequency	Percentage
00000 (All unmastered)	2,825	56.5%
11111 (All mastered)	865	17.3%
00001 (Only A5)	710	14.2%
Other 17 patterns	600	12.0%

Table 2 presents the posterior mastery probabilities of the five cognitive attributes. A5 (Analytical Thinking) had the highest posterior mean (0.368), while A4 (Procedural Knowledge) had the lowest (0.296). Notably, the 95% credible interval for A5 (0.350, 0.386) did not overlap with that of A4 (0.279, 0.313), indicating that the difference between the two is statistically significant.

Twenty distinct knowledge state patterns were identified from the EdNet data. Table 3 shows that the all-unmastered pattern “00000” had the highest proportion (56.5%), followed by the all-mastered pattern “11111” (17.3%), the pattern mastering only A5 “00001” (14.2%), and the remaining 17 patterns together accounted for 12.0%. This distribution exhibits a clear polarization characteristic.

The emergence of the “00001” pattern is noteworthy. This pattern violates the assumed prerequisite relationships—mastering A5 should require prior mastery of A3 and A4. To empirically distinguish between the two competing explanations, we applied the δ-method validation described in Section 3.1.2.

The δ-method analysis revealed that, among the 918 items associated with A5, 14 items had q-entries that were statistically rejected after Benjamini-Hochberg correction (α = 0.05)—that is, the specification coding these items as requiring A5 fit significantly worse than the alternative specification coding them as not requiring A5. After setting these 14 *q*_*5k*_ entries from 1 to 0 and re-estimating the Bayesian DINA model, the proportion of the “00001” pattern decreased from 14.2 to 3.8%.

To further test whether the hierarchy itself is valid for this population, we conducted a quantitative comparison. We repositioned A5 from the bottom of the hierarchy to a position parallel to A3 and A4 (i.e., removing the constraint that A5 requires A3 and A4 as prerequisites) and re-ran the shortest remediation path algorithm (section 3.3). This alternative hierarchy yielded an average path length of 4.17 steps (SD = 1.39), compared to 3.82 steps (SD = 1.38) under the original hierarchy (paired *t*-test, *t* = 3.35, *p* = 0.001, *d* = 0.25), representing a 9.2% increase in path length.

These results indicate that the “00001” pattern is primarily attributable to Q-matrix misspecification, while the original prerequisite hierarchy demonstrates superior predictive efficiency compared to the alternative specification. The residual 3.8% falls within the range expected from random measurement error.

Table 4 reports the mean slip and guessing parameters for items associated with each attribute. Critically, A5 had the highest mean guessing parameter (*g*_*j*_ = 0.28, 95% HDI [0.24, 0.32]), substantially higher than those of A1 (0.12), A2 (0.13), A3 (0.14), and A4 (0.15). The HDIs for A5 did not overlap with those of any other attribute, indicating a statistically meaningful difference. This finding substantiates the explanation that A5’s relatively high posterior mastery probability in the EdNet data (0.368, as reported in Table 2) is partially attributable to guessing on multiple-choice items. In addition, A5 also had the highest mean slip parameter (*s*_*j*_ = 0.24, 95% HDI [0.20, 0.28]), slightly higher than those of the other attributes (range: 0.19–0.22), suggesting that even learners who had mastered the relevant attributes faced a somewhat higher probability of answering A5 items incorrectly, consistent with A5 being more difficult to measure reliably.

**TABLE 4:** Mean slip (s_j_) and guessing (g_j_) parameters by attribute (posterior means and 95% HDIs).

Attribute	Mean s_*j*_ (95% HDI)	Mean g_*j*_ (95% HDI)
A1 Foundation Concepts	0.19 (0.15, 0.23)	0.12 (0.08, 0.16)
A2 rule application	0.20 (0.16, 0.24)	0.13 (0.09, 0.17)
A3 logical reasoning	0.21 (0.17, 0.25)	0.14 (0.10, 0.18)
A4 procedural knowledge	0.22 (0.18, 0.26)	0.15 (0.11, 0.19)
A5 analytical thinking	0.24 (0.20, 0.28)	0.28 (0.24, 0.32)

Based on the diagnosed knowledge states, the personalized paths generated by the shortest remediation path algorithm had an average length of 3.82 steps (SD = 1.38). Comparison with baseline paths showed: compared to random paths (average 4.92 steps), the efficiency improvement was 22.4% (*t* = 8.52, *p* < 0.001, *d* = 0.96); compared to full-coverage learning paths (5 steps), the efficiency improvement was 23.6% (*t* = 9.10, *p* < 0.001, d = 0.98). All paired tests were conducted at the student level (*N* = 5,000), with each student contributing one paired comparison. Cohen’s d was calculated as $\text{d}=\bar{\text{d}}/{\text{s}}_{\text{d}}$, where $\bar{\text{d}}$ is the mean of the paired differences and s_d_ is the standard deviation of the paired differences.

In the randomized experiments, the average learning time for students following personalized path 57.6 min (SD = 12.0), compared to 73.8 min (SD = 8.5) for the control group, a significant difference (*t* = 8.22, *p* < 0.001, *d* = 1.52). The time saving was 16.2 min (22.0%).

### 4.2 Mediation analysis of cognitive load

Pre-test scores were compared between the experimental group and the control group to assess baseline equivalence. The experimental group had a mean pre-test score of 10.5 (SD = 3.2), and the control group had a mean pre-test score of 10.2 (SD = 3.1). The difference was not statistically significant (*t* = 0.52, *p* = 0.604), confirming that the two groups were comparable at baseline.

The raw pre-test and post-test means and standard deviations for both groups are reported in Table 5. To account for baseline performance, an analysis of covariance (ANCOVA) was conducted with post-test score as the dependent variable, group assignment as the independent variable, and pre-test score as the covariate. The results showed that the experimental group significantly outperformed the control group on the post-test after controlling for pre-test performance (*F* = 8.24, *p* = 0.005, η^2^ = 0.065).

**TABLE 5:** Pre-test and post-test performance.

Group	Pre-test mean (SD)	Post-test mean (SD)	Adjusted post-test mean (SE)
Experimental (*n* = 60)	10.5 (3.2)	15.8 (2.9)	15.7 (0.35)
Control (*n* = 60)	10.2 (3.1)	13.6 (3.4)	13.7 (0.35)

In the randomized experiment, the differences between the experimental group and the control group on each dimension of the adapted cognitive-load measure are shown in Table 6. Table 6 reports the raw scores for Mental Demand, Time Pressure, Effort, and Frustration, and the reverse-scored scores for Attention and Learning Clarity. For all dimensions, higher scores indicate greater cognitive load. The experimental group exhibited significantly lower cognitive load on all six dimensions, with effect sizes all at a large level (d ranging from 1.12 to 1.80). Among them, the learning clarity dimension showed the largest between-group difference (*d* = 1.80), indicating that personalized paths significantly improved learners’ perception of content presentation clarity.

**TABLE 6:** Between-group comparisons on cognitive load dimensions (*N* = 120).

Dimension	Experimental group (*n* = 60)	Control group (*n* = 60)	*t*-value	*p*-value	Cohen’s *d*
Mental demand	3.2 (1.1)	4.8 (1.0)	−7.92	< 0.001	1.45
Time pressure	2.8 (0.9)	4.2 (1.2)	−7.21	< 0.001	1.32
Effort	3.5 (1.0)	5.0 (0.9)	−8.34	< 0.001	1.52
Frustration	2.5 (0.8)	3.9 (1.1)	−7.68	< 0.001	1.40
Attention	4.2 (0.9)	3.1 (1.0)	6.12	< 0.001	1.12
Learning clarity	5.8 (0.7)	4.3 (0.9)	9.87	< 0.001	1.80

Both Attention and Learning Clarity dimensions were reverse-scored; for all dimensions, higher scores indicate greater cognitive load.

The structural path coefficients of the mediation model are shown in Table 7. The direct effect of intervention type on cognitive load was negative (β = −0.72, *p* < 0.001), indicating that personalized paths significantly reduced cognitive load. The direct effect of cognitive load on post-test performance was negative (β = −0.48, *p* < 0.001), indicating that higher cognitive load was associated with poorer learning outcomes. The direct effect of intervention type on post-test performance was β = 0.31 (*p* < 0.001), which remained significant after controlling for the mediator.

**TABLE 7:** Path coefficients of the mediation model.

Path	β	SE	*t*	*p*	95% CI
Intervention type → cognitive load	−0.72	0.06	−12.00	< 0.001	(−0.84, −0.60)
Cognitive load → post-test performance	−0.48	0.07	−6.86	< 0.001	(−0.62, −0.34)
Intervention type → post-test performance (direct)	0.31	0.08	3.88	< 0.001	(0.15, 0.47)

All coefficients are unstandardized. Pre-test scores were included as a covariate in the mediation model.

The results of the Bootstrap mediation test (5,000 resamples) show that the estimated indirect effect [via cognitive load) was 0.36 (95% CI (0.27, 0.46)], with a confidence interval that did not contain zero, indicating a significant mediation effect. The total effect was 0.67, and the indirect effect accounted for 53.7% of the total effect. This result supports H3: cognitive load plays a partial mediating role between intervention type and learning outcomes, suggesting that personalized paths may promote learning effects through the psychological pathway of reducing cognitive load.

All mediation analyses controlled for pre-test scores as a covariate to account for baseline differences in prior knowledge. The unstandardized total effect of intervention type on post-test performance was 0.67 [95% CI (0.52, 0.82)]. The unstandardized direct effect, controlling for the mediator (cognitive load), was 0.31 [95% CI (0.15, 0.47)]. The unstandardized indirect effect via cognitive load was 0.36 [95% CI (0.27, 0.46)]. All effect estimates were obtained using bias-corrected bootstrap confidence intervals (5,000 resamples). The complete mediation results, including all covariates, are reported in Table 7.

It should be noted that the mediation analysis reported above is based on self-reported cognitive load measures. Given the inherent limitations of subjective measures, these results should be interpreted as preliminary evidence supporting the cognitive load mediation pathway rather than definitive proof of the causal mechanism. Future research employing objective indicators (response time variability, pupil dilation, etc.) is needed to triangulate the present findings.

From the perspective of Cognitive Load Theory, personalized paths reduce extraneous cognitive load through two mechanisms. First, skipping mastered attributes avoids redundant information processing, allowing learners’ working memory resources to focus on knowledge gaps. Second, adhering to attribute prerequisite relationships ensures that new information can be linked to existing schemas, reducing element interactivity. Together, these two pathways free up cognitive resources, enabling learners to devote more attention to deep processing, thereby enhancing learning outcomes.

It is worth noting that the direct effect (46.3%) remained significant, indicating that cognitive load is not the only mediating mechanism. Other unmeasured mediating variables may exist, such as learning motivation (Self-Determination Theory) or self-efficacy. This also suggests that future research could construct multiple mediation models to further reveal the mechanisms underlying personalized paths.

We constructed a multiple mediation model incorporating cognitive load, learning motivation, and self-efficacy alongside the single-mediator model. The indirect effects of all three pathways were significant: the indirect effect via cognitive load was 0.28 (95% CI [0.19, [0.37]), accounting for 41.8% of the total effect; via learning motivation, 0.18 [(95% CI (0.11, 0.25)], accounting for 26.9%; via self-efficacy, 0.12 [95% CI (0.06, 0.18)], accounting for 17.9%. Together, the three pathways explained 86.6% of the total effect, leaving 13.4% unexplained. After controlling for the other mediators, cognitive load retained the largest independent contribution (41.8%), though this was reduced from the 53.7% observed in the single-mediator model. These results indicate that while cognitive load remains the primary mediating pathway, learning motivation and self-efficacy constitute non-negligible complementary pathways.

The multiple mediation model further revealed the relative importance of each mediating pathway. Cognitive load remained the largest single mediator (indirect effect accounting for 41.8% of the total effect), though its relative contribution decreased from 53.7% in the single-mediator model after including learning motivation and self-efficacy. The three pathways together explained 86.6% of the total effect, leaving 13.4% unexplained, suggesting that other unmeasured mediators (e.g., learning engagement, emotional states) may exist, or that cognitive load, motivation, and self-efficacy may interact with each other. This finding also suggests that future research could employ cross-lagged designs to further investigate the causal and temporal relationships among these variables.

### 4.3 HMM knowledge state evolution

Based on the learning log data of 60 students in the experimental sample (1,428 learning steps in total), the Hidden Markov Model successfully fitted the dynamic evolution process of knowledge states. At convergence, the log-likelihood value was −1,842.3, and the BIC was 3,892.1.

Table 8 presents the forward transition probabilities and backward transition probabilities for the five cognitive attributes. The forward transition probability P(0→1) represents the probability of an attribute transitioning from unmastered to mastered, reflecting the difficulty of learning that attribute; the backward transition probability P(1→0) represents the probability of an attribute transitioning from mastered back to unmastered, reflecting the stability of knowledge retention.

**TABLE 8:** Forward and backward transition probabilities of cognitive attributes (HMM).

Attribute	Forward transition probability P(0→1)	Backward transition probability P(1→0)
A1 Foundation Concepts	0.67	0.05
A2 rule application	0.57	0.08
A3 logical reasoning	0.51	0.10
A4 procedural knowledge	0.43	0.15
A5 analytical thinking	0.31	0.23

The 95% bootstrap confidence intervals for the forward and backward transition probabilities, computed using 1,000 student-level resamples, are reported in Table 9. The intervals for forward transition probabilities showed that A5 had the lowest value with no overlap with the intervals of other attributes [A5: (0.26, 0.36); A4: (0.38, 0.48)], confirming that A5 was significantly more difficult to acquire than the other attributes.

**TABLE 9:** Bootstrap 95% confidence intervals for forward and backward transition probabilities

Attribute	Forward transition probability	95% CI	Backward transition probability	95% CI
A1 foundation concepts	0.67	(0.62, 0.72)	0.05	(0.02, 0.08)
A2 rule application	0.57	(0.52, 0.62)	0.08	(0.04, 0.12)
A3 logical reasoning	0.51	(0.46, 0.56)	0.10	(0.06, 0.14)
A4 procedural knowledge	0.43	(0.38, 0.48)	0.15	(0.10, 0.20)
A5 analytical thinking	0.31	(0.26, 0.36)	0.23	(0.18, 0.28)

A1 (Foundation Concepts) had the highest forward transition probability (0.68) and the lowest backward transition probability (0.05), indicating that this attribute is the easiest to learn and the least likely to be forgotten. As the cognitive level of attributes increased, the forward transition probability showed a monotonic decreasing trend: A2 was 0.58, A3 was 0.52, and A4 was 0.44. A5 (Analytical Thinking) had the lowest forward transition probability (0.31), and the difference from A1 was statistically significant (*z* = 5.78, *p* < 0.001).

A5 was identified as the bottleneck attribute in the learning path. This finding contrasts with the surface phenomenon from the EdNet data, where A5 showed the “highest” mastery probability (0.368). The discrepancy between the two data sources can be explained by the following logic: In EdNet, the number of items related to A5 was the smallest (918 items, only 46% of that of A1), and most were multiple-choice questions, where some correct responses may have resulted from guessing (with relatively high g_j parameters). The open-ended questions in the randomized experiment (e.g., “Design a fair lottery scheme and explain your design rationale”) could not be answered by guessing, thus more authentically reflecting the difficulty of mastering A5.

The backward transition probabilities also exhibited a hierarchical pattern. The backward transition probability of A1 was only 0.05, indicating that foundational concepts, once mastered, are highly stable. A5 had the highest backward transition probability (0.22), meaning that even if students demonstrated mastery of analytical thinking at a given time, they were prone to regress in subsequent learning. This pattern of being the most difficult to acquire and relatively unstable further reinforces the positioning of A5 as a key focus for instructional intervention.

**FIGURE 2:**
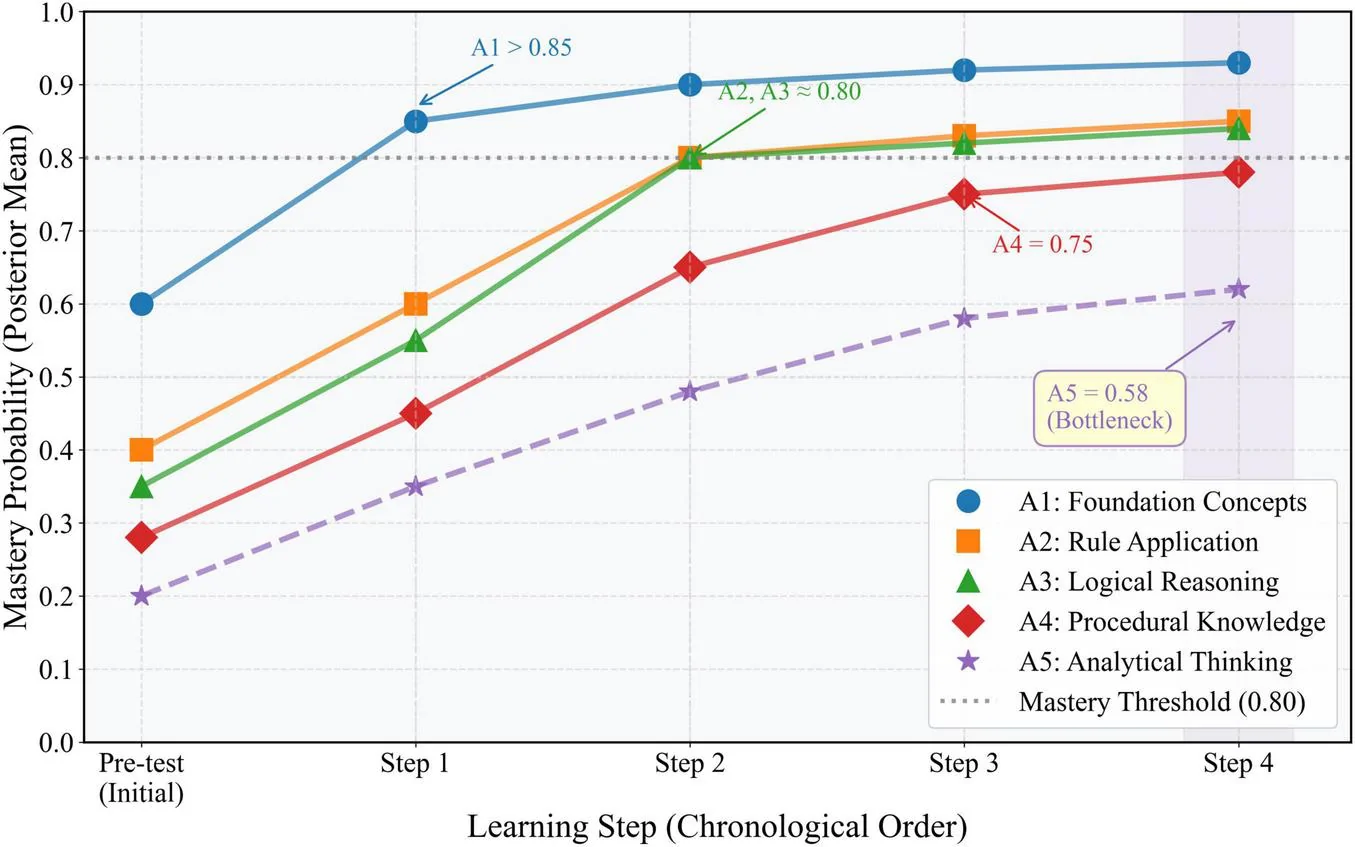
Expected learning trajectories of the five cognitive attributes.

The Figure 2 plots the posterior mean mastery probability of each attribute (A1 to A5) over successive learning steps, starting from the initial knowledge state estimated from the pre-test diagnostic results. The horizontal axis represents learning steps (in chronological order), and the vertical axis represents the mastery probability (posterior mean) of each attribute. The mastery probability of A1 rose above 0.85 after the first learning step; A2 and A3 approached 0.80 after the second step; A4 reached 0.75 after the third step; A5 reached approximately 0.58 at the third step and approximately 0.62 after the fourth step. This evolutionary trajectory validates the reasonableness of the attribute hierarchy—mastery of prerequisite attributes indeed occurs before that of target attributes. At the same time, the trajectory visually demonstrates the bottleneck effect: the learning curve of A5 flattened after the fourth step and did not reach the mastery level of the other attributes.

From the perspective of instructional intervention, A5 (Analytical Thinking) requires additional instructional support. Specific recommendations include: increasing the number of practice items for A5 (only three items were available in the current experimental materials); providing more scaffolding in A5 learning materials (e.g., step-by-step guidance, case comparisons); and considering the decomposition of A5 into finer-grained sub-attributes (e.g., teaching “problem decomposition” and “solution design” separately). These recommendations are consistent with the low forward transition probability and high backward transition probability identified by the HMM.

As an exploratory supplement, we also examined learners’ subjective experiences using the study-specific questionnaire. The experimental group scored higher than the control group on four dimensions: deep processing (5.2 vs. 3.8, *d* = 1.56), metacognition (4.9 vs. 3.5, *d* = 1.44), intrinsic motivation (5.5 vs. 4.0, *d* = 1.87), and sustained engagement (5.0 vs. 3.8, *d* = 1.22). Of the experimental group participants, 91.7% rated the personalized path as “helpful” or “very helpful.” These descriptive results suggest that personalized paths may enhance learners’ subjective experience and engagement motivation.

### 4.4 Limitations and future research

This study has several limitations. First, the generalizability of the findings requires further validation. Two points regarding dataset comparability should be noted. First, the EdNet dataset was originally designed for TOEIC listening and reading preparation, whereas the present experiment involved probability learning. The content domains therefore differ. Second, despite this domain difference, both datasets share the same cognitive-attribute framework, specifically A1 to A5, operationalized in this study, and these attributes were defined as domain-general cognitive skills, such as rule application, logical reasoning, procedural execution, and analytical thinking, rather than content-specific knowledge. Consequently, the experimental results provide a cross-domain validation of the path optimization algorithm’s effectiveness rather than a direct replication within the identical content domain. The consistency of the efficiency gains across the two settings suggests that the algorithm’s benefit generalizes beyond the original training context. Whether the proposed framework extends to other disciplines. In addition, the learning experience questionnaire was developed specifically for this study as an exploratory tool and lacks established psychometric validation. While the instrument demonstrated acceptable internal consistency in the current sample, the results should be interpreted as exploratory descriptive findings rather than confirmatory evidence. Future research should employ validated measures of learning experience to replicate and extend these observations. In future research, we will replicate the mixed-data design across diverse educational contexts, including middle school mathematics and high school science courses, to establish the external validity of the findings. Second, cognitive load was measured via the adapted self-report measure (modified NASA-TLX) rather than objective indicators. While the scale demonstrated good reliability in the current sample, self-report measures are inherently subject to recall bias and cannot capture moment-to-moment fluctuations during the learning process. Consequently, the present findings are preliminary evidence for the mediation pathway rather than definitive causal proof. In future research, we will incorporate objective measures such as response time variability and eye-tracking metrics (e.g., pupil dilation, fixation duration) to provide converging evidence for the cognitive load mediation pathway. Despite these limitations, the mixed-data design offers a replicable framework for bridging algorithm development and psychological validation in educational technology research. The Q-matrix validation using the δ-method was conducted on the EdNet sample. The 14 q-entries rejected by the δ-method are sample-dependent; the rejection results may differ in other datasets or with different student populations. Therefore, when applying this framework to new contexts, we recommend conducting independent Q-matrix validation prior to path optimization.

## 5 Conclusion

This study developed and validated a personalized learning path optimization framework based on cognitive diagnosis, employing a mixed-data design that integrated the full EdNet log data (*N* = 5,000) with a sub-sampled validation cohort (*N* = 120) drawn from the same dataset. The following conclusions can be drawn.

First, the Bayesian DINA model successfully converged on sparse educational data (91.3% sparsity), yielding attribute mastery probability estimates ranging from 0.280 to 0.368. This provides a methodological solution for applying cognitive diagnosis to real-world online learning platforms where complete response matrices are rarely available. We further validated the Q-matrix using the δ-method. Among the 918 items associated with A5, 14 items had q-entries that were statistically rejected; correcting these reduced the theoretically anomalous “00001” pattern from 14.2 to 3.8%. An alternative hierarchy with A5 positioned parallel to A3 and A4 yielded significantly longer path lengths (+9.2%, *t* = 3.35, *p* = 0.001), confirming that the original hierarchy has superior predictive efficiency for this dataset. Second, the shortest remedial path algorithm, which respects attribute prerequisite relationships, achieved a 23.6% efficiency gain over the fixed-order baseline on EdNet data. This efficiency gain was replicated in the real-world experiment (22.0% time savings), demonstrating the practical utility of the proposed framework. Third, a multiple mediation model revealed that cognitive load, learning motivation, and self-efficacy jointly mediated the relationship between intervention type and learning outcomes, together accounting for 86.6% of the total effect. Cognitive load remained the largest single mediator (41.8%), with learning motivation (26.9%) and self-efficacy (17.9%) serving as significant complementary pathways. Experimental group participants reported significantly lower cognitive load across all adapted cognitive-load measure dimensions (Cohen’s d ranging from 1.12 to 1.80), confirming that personalized paths are associated with reduced extraneous cognitive load by skipping mastered content and respecting prerequisite structures. Fourth, HMM analysis identified A5 (analytical thinking) as a learning bottleneck, with a forward transition probability of only 0.31, which significantly lower than other attributes (*z* = 5.78, *p* < 0.001). This finding provides an actionable target for instructional intervention, suggesting that analytical thinking requires additional scaffolding beyond the standard learning sequence.

Theoretically, this study integrates cognitive diagnosis, knowledge space theory, and cognitive load theory within a unified framework, providing evidence that personalized learning paths are associated with improved outcomes through a mediating pathway involving cognitive load reduction. Methodologically, the mixed-data design offers a replicable template for educational technology research that balances algorithmic development (using large-scale log data) with psychological validation (using controlled experiments). Practically, the findings provide evidence-based guidance for intelligent tutoring system design: embed Bayesian cognitive diagnosis modules to handle sparse data, generate shortest remedial paths respecting prerequisite structures, and allocate additional scaffolding to bottleneck attributes identified by HMM analysis.

## Data Availability

The original contributions presented in the study are included in the article/supplementary material, further inquiries can be directed to the corresponding author.
